# Nrf2 antioxidant pathway suppresses Numb-mediated epithelial–mesenchymal transition during pulmonary fibrosis

**DOI:** 10.1038/s41419-017-0198-x

**Published:** 2018-01-23

**Authors:** Zhihui Zhang, Jiao Qu, Cheng Zheng, Panpan Zhang, Wencheng Zhou, Wenhui Cui, Xiaoting Mo, Liucheng Li, Liang Xu, Jian Gao

**Affiliations:** 1 The First Affiliated Hospital of Anhui Medical University, Hefei, Anhui 230022 China; 2 The Second Affiliated Hospital of Dalian Medical University, Dalian, Liaoning 116023 China; 3 School of Pharmacy, Dalian Medical University, Dalian, Liaoning 116044 China; 4 School of Pharmacy, Anhui Medical University, Hefei, Anhui 230032 China

## Abstract

Epithelial mesenchymal transition (EMT) is a key progression that promotes pulmonary fibrosis (PF). Numb, a phosphotyrosine-binding domain (PTB) protein, is implicated with EMT. Nuclear factor erythroid 2-related factor2 (Nrf2) and its downstream proteins heme oxygenase-1 (HO-1) and NAD(P)H: quinone oxidoreductase 1 (NQO1) constitute an important pathway of antioxidant defense signal for protecting against PF. It remains elusive whether Nrf2 antioxidant pathway and Numb have a potential relationship in EMT-mediated PF. Here, we observed the effects of Nrf2 pathway and Numb on bleomycin(BLM)-induced PF in Nrf2-knockout (Nrf2^−/−^) and wild-type (WT) mice. Meanwhile, rat type II alveolar epithelial cells line (RLE-6TN) and human epithelial cells line (A549) were both treated with an Nrf2 activator sulforaphane (SFN), or transfected siRNAs of Nrf2 and Numb to unravel roles of Nrf2 pathway, Numb and the link between them on transforming growth factor β1 (TGF-β1)-induced EMT. We found BLM-induced lung fibrosis were more severe in Nrf2^−/−^ mice compared to WT mice with reduced expressions of HO-1 and NQO1. Numb was enhanced with down-regulated expressions of Nrf2 in BLM groups and further increased in Nrf2^−/−^ groups. In vitro, given exogenous TGF-β1 on RLE-6TN and A549 up-regulated Numb expressions, accompanied with down-regulations of Nrf2 and its target proteins HO-1 and NQO1. Transfected with Nrf2 and Numb siRNAs further aggravated and relieved the progression of EMT, respectively. Inversely, activating Nrf2 pathway by SFN reduced the expression of Numb and EMT-related protein. Moreover, Numb deficiency by siRNA relieved the protection of activating Nrf2 against EMT. In conclusion, activating Nrf2 antioxidant pathway suppresses EMT during PF via inhibiting the abnormal expression of Numb. These findings provide insight into PF pathogenesis and a basis for novel treatment approaches.

## Introduction

Pulmonary fibrosis (PF) is a chronic interstitial lung disease typically characterized by excessive production and deposition of extracellular matrix (ECM) and remodeling of abnormal lung tissue structure^1^. According to statistics, the prevalence of PF is 1.6–1.7/10000^2^, however, the pathogenesis of PF is not well understood, and new therapeutic strategies for its prevention and treatment are urgently needed.

Epithelial–mesenchymal transition (EMT) serves as one of the most important avenues for the production of mesenchymal cells and excessive secretion of ECM, which promotes the development of PF^3^. It is a process performed by the loss of epithelial adhesion protein E-cadherin (E-cad) and the acquisition of interstitial cell marker α-smooth muscle actin (α-SMA). Myofibroblasts derived from epithelial cells by EMT exhibit abnormal proliferation and ECM overproduction, leading to the development of PF^4^. However, there is still lack of comprehensive understanding of molecular mechanisms of EMT during the development of PF.

Latest evidence has shown that the RNA expression of Numb occurs frequently in idiopathic pulmonary fibrosis and chronic obstructive pulmonary disease patients^5^. It is a cell fate determinant during development that is also expressed in mature tissues, acting as a negative regulator of NOTCH, WNT, and Hedgehog signaling pathways to control cell differentiation, migration, and tissue regeneration^6^. Many studies have confirmed Numb protein has a close contact with EMT^7^. Recent research shows that Numb binds to the NVYYY domain of E-cad through its phosphotyrosine-binding domain to induce intracellular localization of E-cad and promote cell adhesion in Madin Darby canine kidney cells^8^. Moreover, Numb suppresses E-cad expression to induce EMT in response to transforming growth factor (TGF-β1) signaling in renal fibrosis^9^, but inhibits EMT by antagonizing Notch signaling in triple-negative breast cancer^10^. However, the role of Numb in the development of EMT during PF has not been clarified yet.

Oxidative stress (OS) is one of the most important inducers in the pathogenesis of PF^11^. It is a phenomenon trending to be oxidized when body subjected to external stimulus or the internal environment disorder. Nuclear factor erythroid 2-related factor 2 (Nrf2) is a significant transcription factor for regulating OS^12^ by activating downstream antioxidant proteins including heme oxygenase (HO-1) and NAD(P)H:quinone oxidoreductase (NQO1)^13^. We previously showed that Nrf2 blocked EMT progression in a bleomycin (BLM)-induced PF model^14^. In this study we further explore whether HO-1 and NQO1 are involved in EMT-induced PF when Nrf2 is activated. Meanwhile, associated study has reported that Numb acts as a protective molecule on OS agent puromycin aminonucleoside-induced apoptosis in human renal tubular epithelial cells^15^. Nonetheless, the link between OS and Numb is rarely reported in PF, and the potential contact between Numb and Nrf2 antioxidant pathway has not been verified yet.

In this research we separately constructed BLM-induced PF models in vivo and TGF-β1-induced EMT models in vitro to investigate whether Numb could participate in the progression of EMT-induced PF and the unknown connection between Nrf2-dependent antioxidant pathway and Numb.

## Results

### Expression of Numb and Nrf2 antioxidant pathway in BLM-induced PF models

**Fig. 1:**
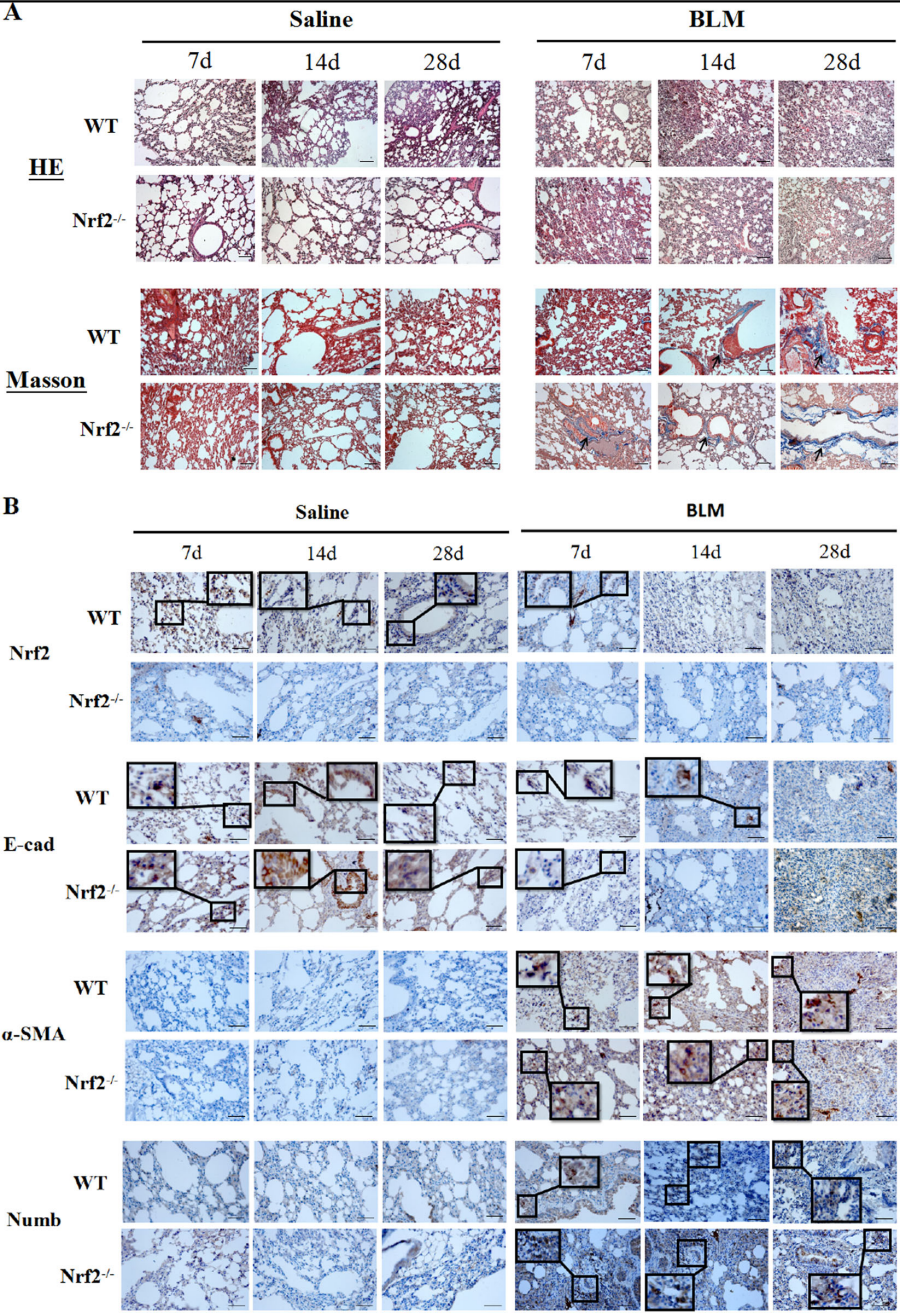
The expressions of Nrf2, Numb and EMT in BLM-induced PF. C57 mice were treated with an endotracheal injection of BLM (4.5 mg/kg) for 7, 14 and 28 days. **a** The lung sections from each mouse were stained with H&E staining and Masson’s trichrome staining to observe the histopathological structure and collagen accumulation respectively. Scale bars represent 100 μm. (The black arrows indicated the collagen expression). **b** Relative protein expressions of Nrf2, Numb, α-SMA and E-cad were measured by IHC. Scale bars represent 50 μm. Boxes were used to enlarge positive tissue, which expressed target proteins

**Fig. 2:**
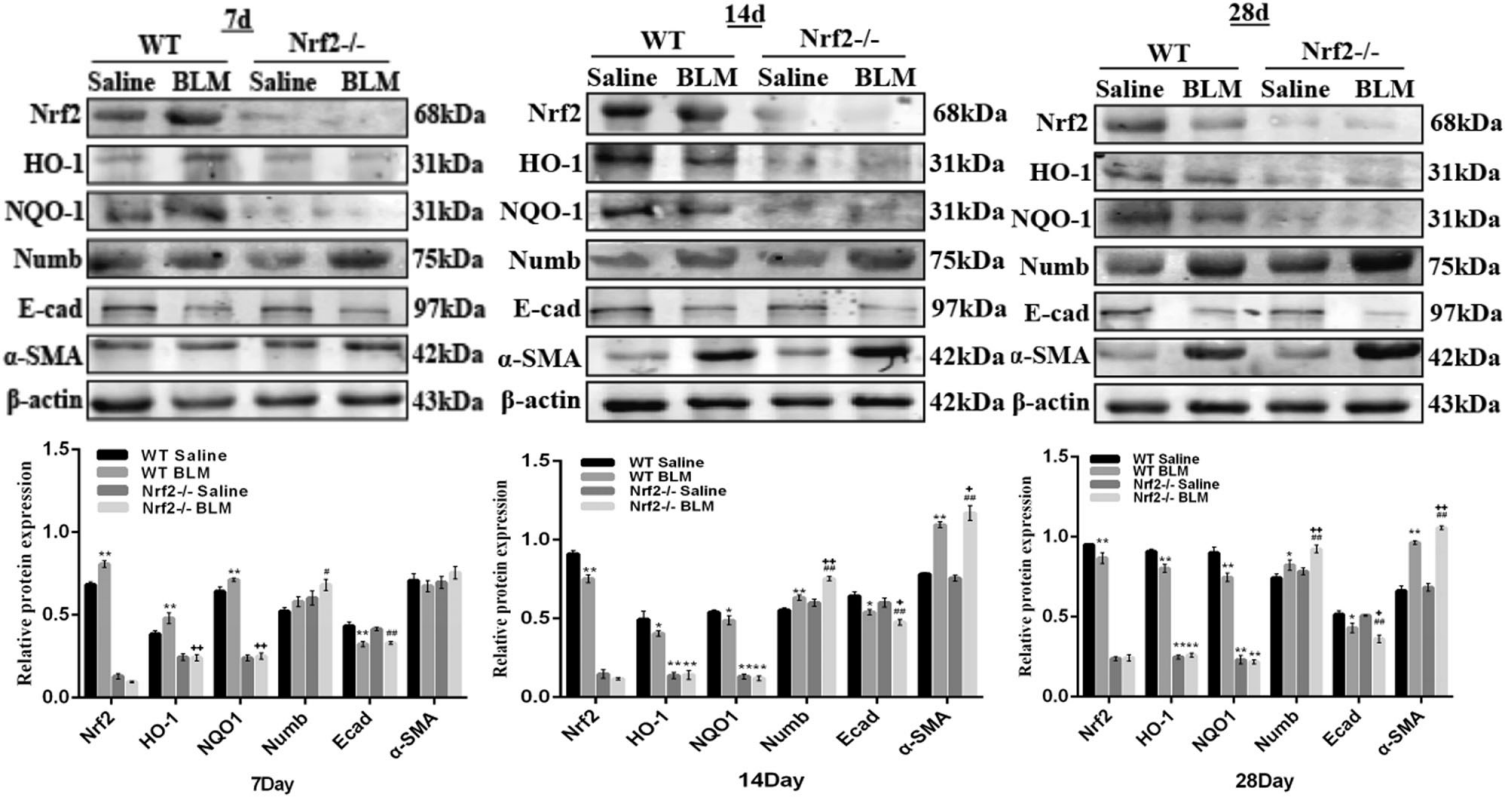
The expression of Nrf2 antioxidant pathway, Numb and EMT in BLM-induced PF. Right lung tissues of mouse were subjected to Western blot analysis for Nrf2, HO-1, NQO1, Numb, α-SMA and E-cad. The representative bands were obtained from different gels for repeated experiments. After densitometric analysis, β-actin was used as an internal reference for relative quantification. Data represent the mean ± SD (*n* = 3 per group), *P < 0.05, **P < 0.01 compared with WT saline group; ^#^*P* < 0.05, ^##^*P* < 0.01 compared with Nrf2^−/−^ saline group; ^+^*P* < 0.05, ^++^*P* < 0.01 compared with WT BLM group

In vivo PF model, we first observed the abnormal pathologic changes after BLM administration. Through H&E and Masson’s trichrome staining, we found interalveolar septum were thickened, the normal structures of the alveoli were destroyed, accompanied with the infiltration of a large number of inflammatory cells and the deposition of ECM in the model group, and these phenomenon were more severe in Nrf2 knockout mice. Staining results reminded that the PF models were successfully established and Nrf2 played a protective role in PF (Fig. 1a). To further explore the role of Numb and Nrf2 antioxidant pathway in the pathogenesis of PF in vivo models, several related proteins were respectively measured by immunohistochemistry (IHC) and Western blot. The expression of epithelial cell adhesion marker E-cad was further reduced in BLM groups with up-regulating expressions of Numb and mesenchymal cell marker α-SMA (Fig.1b). We also evaluated the expressions of Numb, Nrf2, and its downstream effectors HO-1 and NQO1 in mouse lung tissue by western blotting (Fig. 2). Nrf2, HO-1, and NQO1 were stressfully up-regulated on day 7. However, HO-1 and NQO1 were down-regulated in the Nrf2^−/−^ and BLM groups. At the same time, Numb expression was increased on days 7, 14, and 28 in BLM-treated WT and Nrf2^−/−^ groups as compared to the untreated control, with a more robust increase observed in Nrf2 knockout mice. These results suggest that Nrf2, HO-1, NQO1, and Numb participated in the progression of BLM-induced PF and Numb expression is modulated by Nrf2 antioxidant signaling. Moreover, the observed decrease and increase in E-cad and α-SMA levels, respectively, indicate that EMT was enhanced over time (Fig. 2).

### Increased expression of Numb in TGF-β1-induced EMT on RLE-6TN and A549 cells

**Fig. 3:**
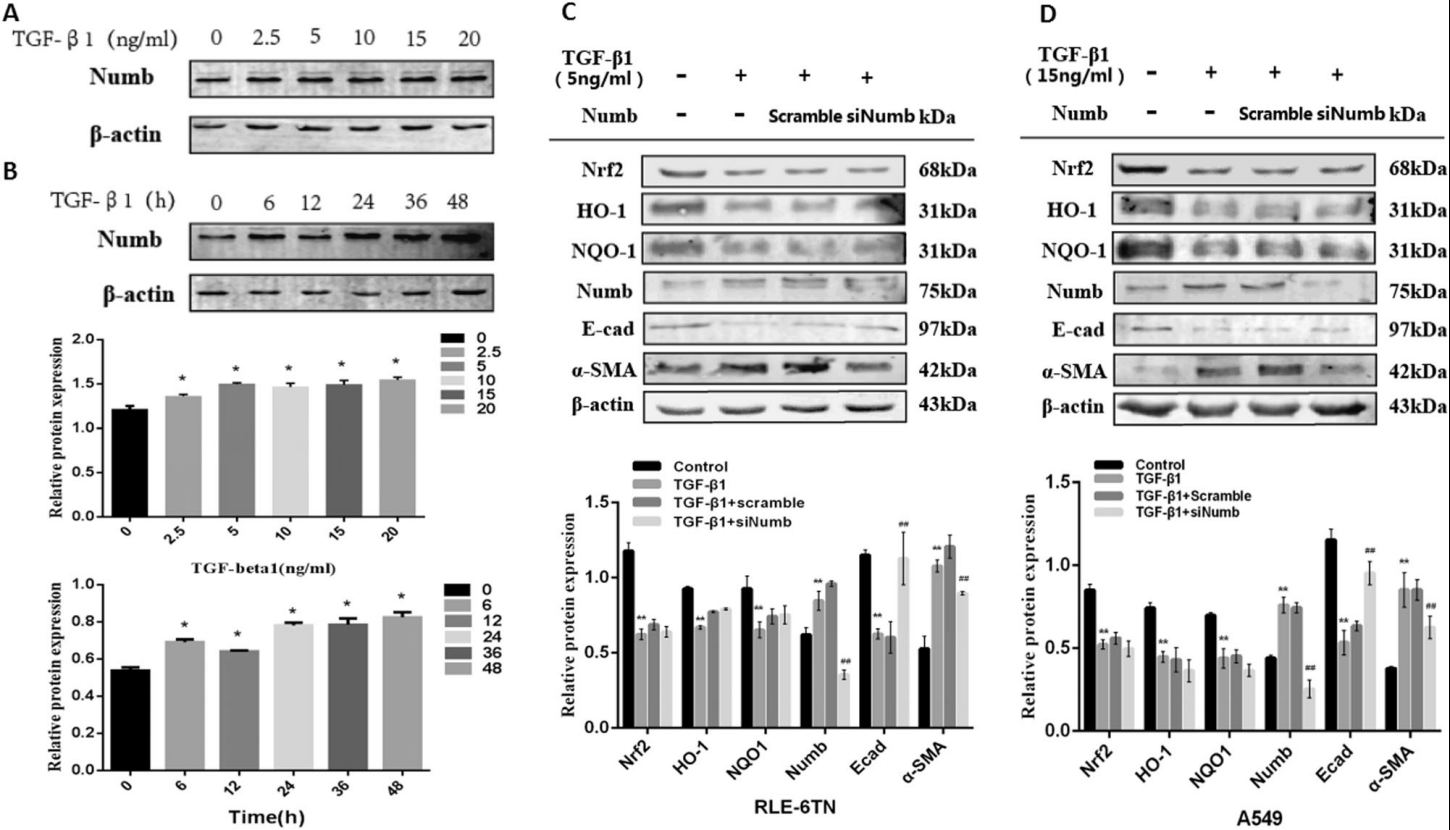
TGF-β1 induced Numb expression in RLE-6TN and A549. **a** RLE-6TN cells were stimulated by TGF-β1 (0-20 ng/ml) for 24 h, then collecting cell total proteins for Western Blot analysis. **b** RLE-6TN cells were treated with 5 ng/ml TGF-β1 for different time points (0–48 h). β-actin was used as an internal reference for relative quantification. Data represent the mean ± SD (*n* = 3 per group), *P < 0.05, **P < 0.01 compared with vehicle group. Numb siRNA was transfected in RLE-6TN (**c**) and A549 (**d**) before stimulated with TGF-β1 for 24 h, then cell proteins were collected and the expressions of Nrf2, HO-1, NQO1, Numb, α-SMA and E-cad were detected by Western blot. The representative bands were obtained from different gels for repeated experiments. After densitometric analysis, β-actin was used as an internal reference for relative quantification. Data represent the mean ± SD (*n* = 3 per group), *P < 0.05,**P < 0.01 compared with vehicle group; ^#^*P* < 0.05, ^##^*P* < 0.01 compared with TGF-β1 group

In the previous part of the experiments, we observed Numb was up-regulated in fibrosis models with the emergence of EMT. Thus we speculated whether Numb could promote the process of EMT. Different concentrations of TGF-β1 were first treated on RLE-6TN for 24 h. TGF-β1 induced the expression of Numb in RLE-6TN cells in a dose-dependent manner (Fig. 3a). Numb protein level began to increase at 2.5 ng/mL of TGF-β1 treatment, and the level reached the plateau at 20 ng/mL of TGF-β1 treatment. Based on these data, RLE-6TN cells were incubated with 5 ng/mL of TGF-β1 for various time periods. As shown in Fig.3b, TGF-β1 induced the expression of Numb in a time-dependent manner in RLE-6TN, with a significant increase of the Numb protein level initially detected at 24 h, and the level remained at a plateau till 48 h. Then, TGF-β1 was used to construct EMT model in vitro. We silenced Numb by small interference RNA (siRNA) on RLE-6TN and A549 before TGF-β1 stimulation [14, 16], the process of EMT in these two cell lines had been alleviated with the increase of epithelial cell marker E-cad and the decrease of mesenchymal cell marker α-SMA (Fig. 3c, d). The above results suggested Numb may be a positive participator protein involving in EMT-induced PF.

### Role of Nrf2-dependent antioxidant pathway on TGF-β1-induced EMT in RLE-6TN and A549

**Fig. 4:**
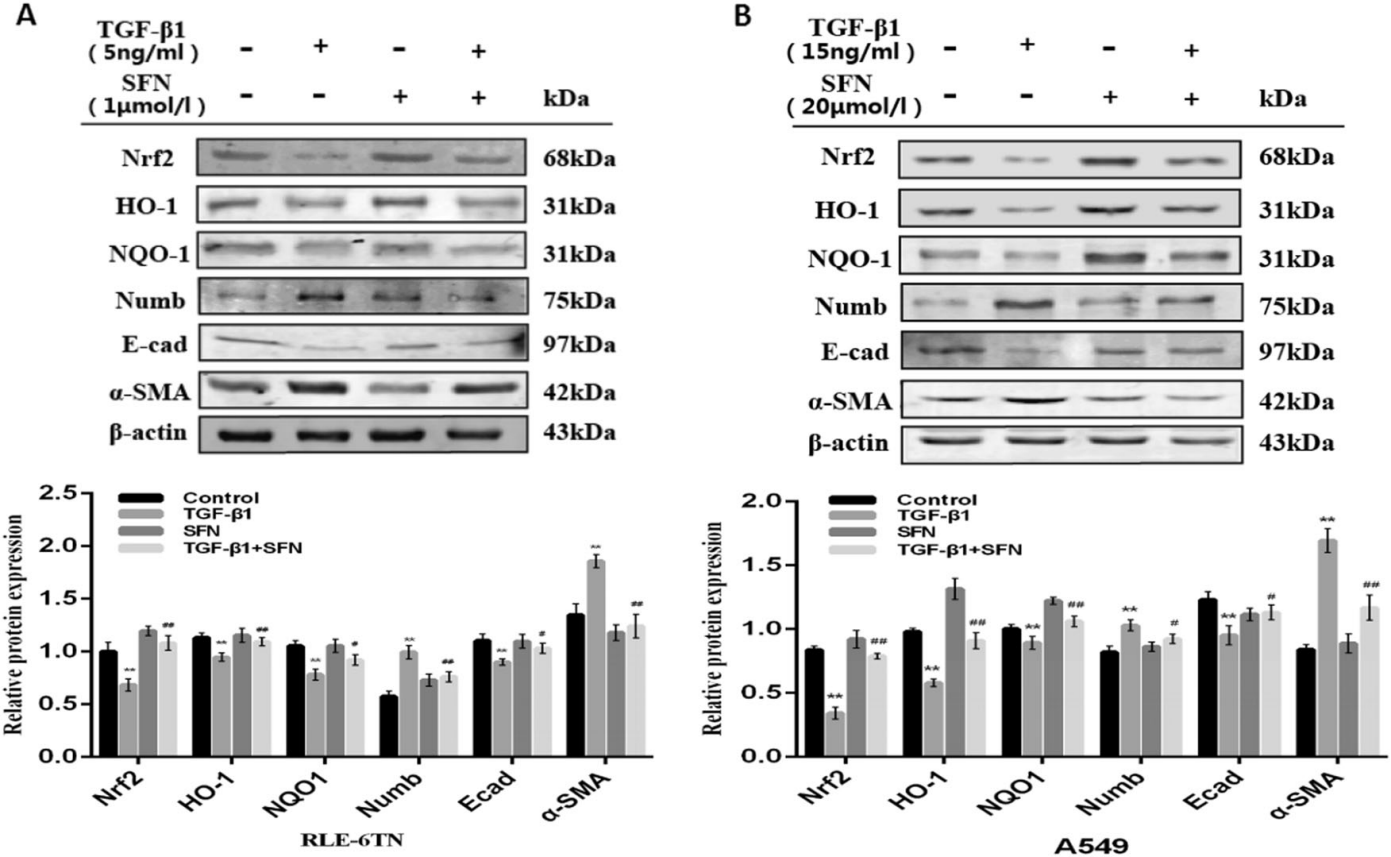
Activation of Nrf2 antioxidant pathway alleviated TGF-β1-induced EMT in RLE-6TN and A549. SFN was pre-treated in RLE-6TN (**a**) and A549 (**b**) for 24 h before stimulated with TGF-β1. After TGF-β1 stimulating for 24 h, all cell protein were collected and the expressions of Nrf2, HO-1, NQO1, Numb, α-SMA and E-cad were detected by Western blot. Data represent the mean ± SD (*n* = 3 per group), *P < 0.05, **P < 0.01 compared with vehicle group; ^#^*P* < 0.05, ^##^*P* < 0.01 compared with TGF-β1 group

**Fig. 5:**
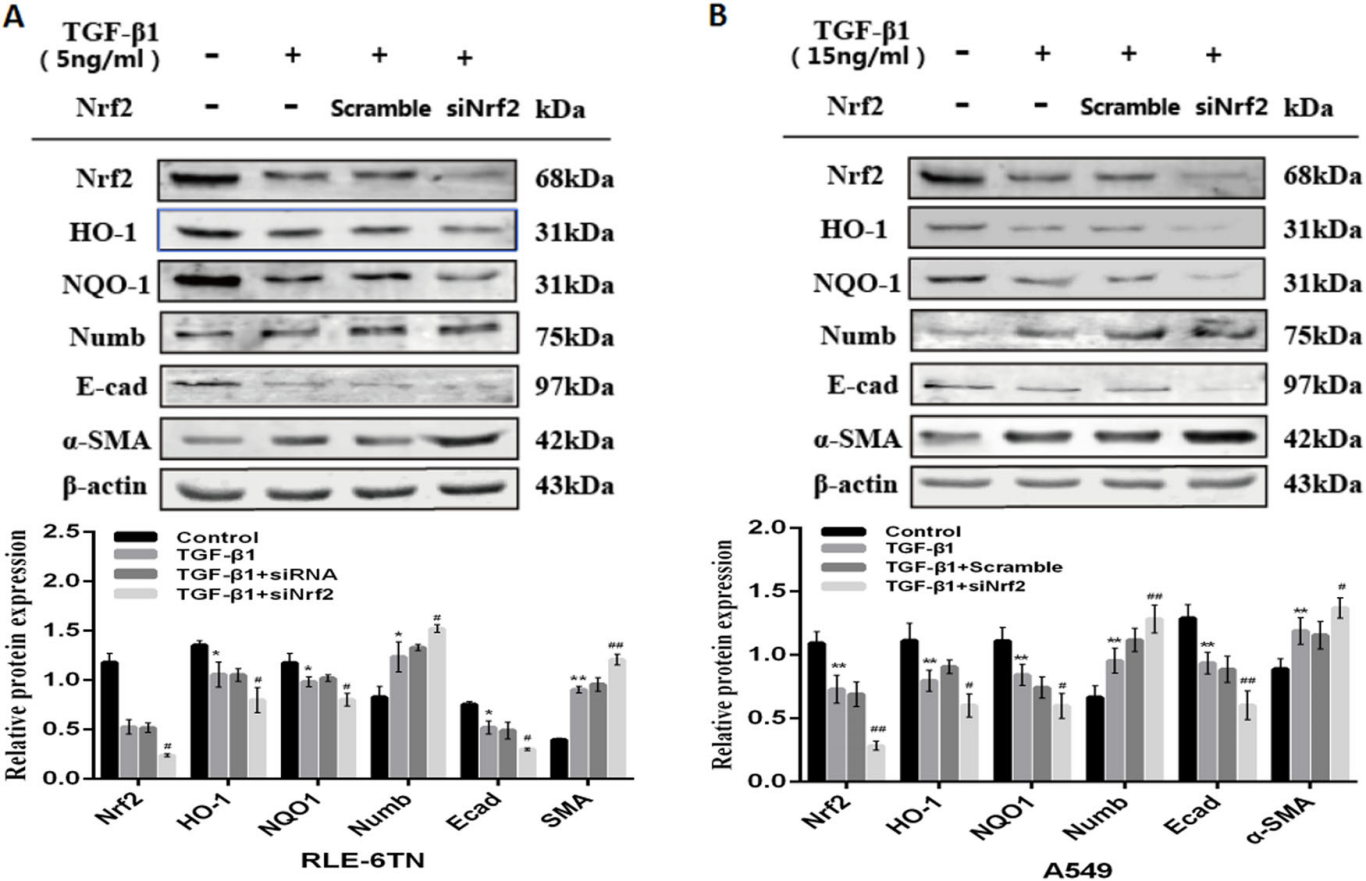
Inhibiting Nrf2 antioxidant pathway by silencing Nrf2 aggravated TGF-β1-induced EMT in RLE-6TN and A549. Nrf2 siRNA was transfected in RLE-6TN (**a**) and A549 (**b**) before stimulated with TGF-β1 for 24 h, then cell proteins were collected and the expressions of Nrf2, HO-1, NQO1, Numb, α-SMA and E-cad were detected by Western blot. The representative bands were obtained from different gels for repeated experiments. After densitometric analysis, β-actin was used as an internal reference for relative quantification. Data represent the mean ± SD (*n* = 3 per group), *P < 0.05, **P < 0.01 compared with vehicle group; ^#^*P* < 0.05, ^##^*P* < 0.01 compared with TGF-β1 group

**Fig. 6:**
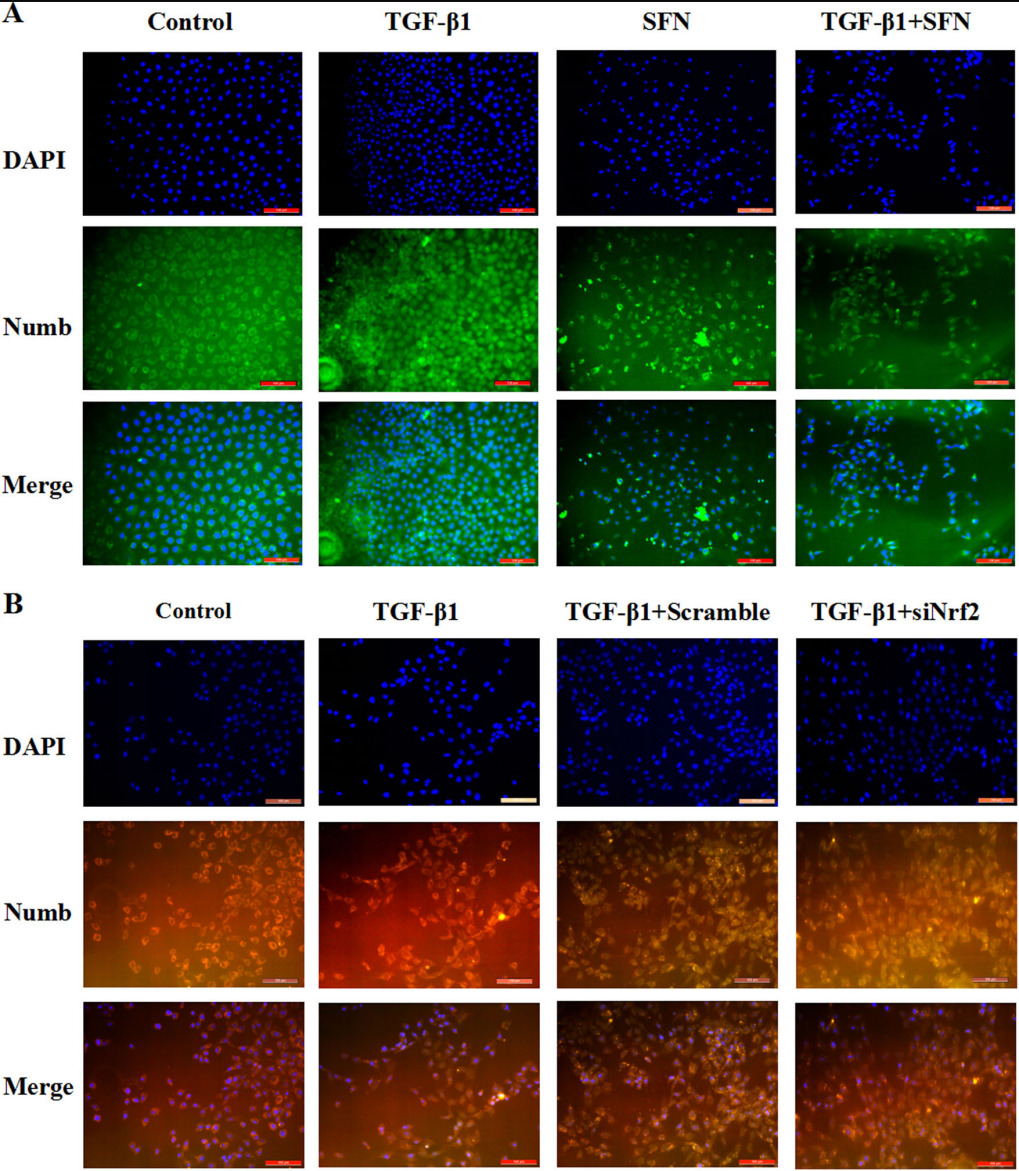
Sulforaphane inhibits the abnromal expression of Numb induced by TGF-β1. **a** Dual immunofluorescence in RLE-6TN cells for Numb (green) and nuclei (blue) was visualized by DAPI staining. Scale bars represent 100 μm. **b** Dual immunofluorescence in RLE-6TN cells for Numb (orange) and nuclei (blue) was visualized by DAPI staining. Scale bars represent 100 μm

In vitro experiment, RLE-6TN and A549 were treated with Nrf2 activator Sulforaphane (SFN) before TGF-β1 stimulation. After 24 h stimulation, we extracted the total cell protein and analyzed by western blot. We separately gave 5 ng/mL TGF-β1, 1 μmol/L of SFN^14^ on RLE-6TN and 15 ng/mL TGF-β1^16^, 20μmol/L^17^ SFN on A549. In both cell lines, epithelial cell adhesion marker protein E-cad was reduced and the interstitial cell marker protein α-SMA was increased in TGF-β1-stimulation group compared to vehicle group with the down-regulated levels of Nrf2, HO-1 and NQO1. But pre-treatment with SFN on RLE-6TN and A549, we found SFN alleviated TGF-β1-induced EMT, with a up-regulation of E-cad and a down-regulation of α-SMA. Additionally, SFN prompted the expressions of Nrf2, HO-1 and NQO1, and reduced the expression of Numb (Fig. 4a, b). Moreover, when two cell lines were transfected with Nrf2 siRNA before TGF-β1 treatment, the expressions of HO-1 and NQO1 were reduced and we observed TGF-β1-induced EMT was aggravated accompanied with augment on the expression of Numb (Fig. 5a, b). At the same time, the expressions of Numb respectively up-regulated and down-regulated by TGF-β1 and SFN stimulating were observed by immunofluorescence, and further increased when Nrf2 was silenced before TGF-β1 treating. Moreover, we magically found the expression of Numb was up-regulated in nucleus and cytoplasm after TGF-β1 stimulating, but pre-treatment with Sulforaphane before the simulation of TGF-β1 could significantly reduce the expression of Numb in nucleus and cytoplasm, and the most of Numb expressing in cytomembrane (Fig. 6a, b). These data demonstrate that activating Nrf2 antioxidant pathway attenuates TGF-β1-induced EMT and the expressions of Numb in RLE-6TN and A549.

### Pharmacological activation of Nrf2 antioxidant pathway reduced EMT via down-regulating the expression of Numb

**Fig. 7:**
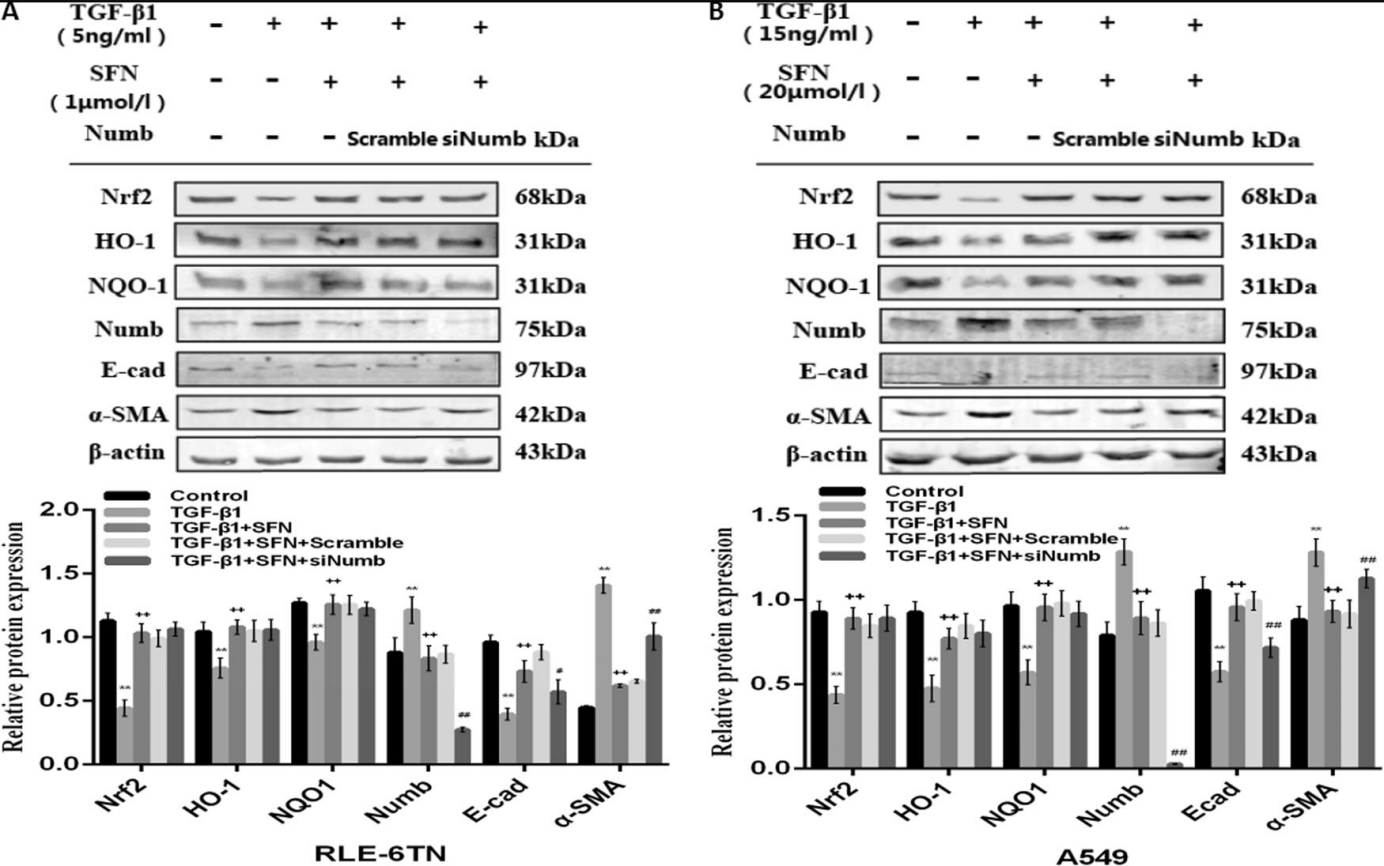
Nrf2 antioxidant pathway inhibited the progression of EMT via suppressing the expression of Numb. Before SFN stimulation on RLE-6TN (**a**) and A549 (**b**), Numb siRNA was transfected in two cell lines. Finally we treated two cell lines with TGF-β1 stimulation. After densitometric analysis, β-actin was used as an internal reference for relative quantification. Data represent the mean ± SD (*n* = 3 per group), *P < 0.05, **P < 0.01 compared with vehicle group; ^+^*P* < 0.05, ^++^*P* < 0.01 compared with TGF-β1 group; ^#^*P* < 0.05, ^##^*P* < 0.01 compared with TGF-β1 + SFN group

To further investigate whether Nrf2 antioxidant pathway block the process of EMT via Numb, we translated siRNA of Numb in A549 and RLE-6TN, and SFN was given in two cell lines before TGF-β1 stimulation. Then, we collected total cell protein for western blot analysis. Surprisingly, the protective effect of SFN against EMT in silenced Numb group was mitigated compared with un-silenced Numb group, which was reflected on the up-regulation of α-SMA and down-regulation of E-cad (Fig. 7a, b). These results suggested us Numb took participate in the process of Nrf2 antioxidant pathway ameliorating the progression of EMT.

## Discussion

PF is a fatal interstitial lung disease characterized by ECM over-deposition and structural remodeling of lung tissue. The etiology of PF formation is very complex, and the specific mechanism is not clear. The understanding of the pathogenesis of PF is not sufficient, resulting in lacking effective therapy. Therefore, exploring the pathogenesis of PF and looking for new targets to combat PF are urgently needed, which possesses great significance for clinical treatment. In this study, we demonstrated that abnormal expression of Numb could involved in the progression of EMT during PF. However, activating Nrf2 antioxidant pathway could effectively alleviate the development of EMT by regulating the unusual expression of Numb. These findings may be helpful in improving our understanding of the pathogenesis of PF and promoting the development of effective therapies.

In the early stage of PF, inflammatory cells, epithelial cells, and endothelial cells in the lungs are damaged by exogenous material release cytokines such as TGF-β1 and other chemokines that promote wound and vascular repair. A large number of stromal cells such as fibroblasts, endothelial cells and epithelial cells are induced by TGF-β1 to differentiate into myofibroblasts, leading to abnormal proliferation and secretion of ECM^18–20^. Therefore, EMT serves as an important source of MFbs, playing a crucial role in secreting excessive ECM and promoting the development of PF^21^. In vivo experiments, a large amount of collagen was observed in BLM-induced PF mice model by Masson’s trichrome staining. Moreover, the protein level changes of E-cad and α-SMA detected by Western blot and IHC further suggested EMT involves in BLM-induced PF. Based on previous studies, TGF-β1 plays an important role in promoting EMT during PF^22^, therefore we treated RLE-6TN and A549 cells with exogenous TGF-β1 to build EMT models in vitro. Although A549 is lung cancer cell, it is so similar to type II alveolar epithelial cells that widely used for the study of EMT ^23–25^.

In addition, we investigated a membrane associated protein Numb that was involved in the process of EMT in PF. Numb usually takes part in EMT-mediated tumor and renal fibrosis by early researches^9, 10, 26–28^. But the influence of Numb in PF still has not been explored. Recent study has shown Numb is augmented by TGF-β1 stimulation in renal fibrosis^9^. Therefore, we conducted TGF-β1 to stimulate RLE-6TN and A549 cells, the expression of Numb was increased, however the degree of EMT was alleviated when Numb was silenced by siRNA. In vivo we also received the similar trend, which the expressions of Numb were increased in BLM groups and more serious in Nrf2 knockout mice. The avenue of Numb how to effect the process of EMT in PF remained to be deeply investigated by next study. We initially speculated that Numb could affected the expression of E-cad in the epithelium to promoting EMT. Ding et al. reported that ectopic Numb induces E-cad adhesion dissolution by AP-2-dependent endocytosis in NRK52E cells^9^. But the approach of Numb affecting the expression of E-cad in EMT-involved PF requires more studies to explore.

OS is a crucial inducer of PF. The mechanism of OS promoting the PF mainly includes the following three pathways^29, 1^: Forming direct injury on AECs and promoting apoptosis^2^; tumor necrosis factor α, interleukin, nuclear transcription factor-kB are induced to over-express and release a large number of inflammatory factors^3^; direct-activating TGF-β1. The redox-sensitive transcription factor Nrf2 acts as a regulator of antioxidant enzyme and defensive protein involving in the progress of OS. Our previous research has found that Nrf2 alleviates EMT in PF by suppressing Snail^14^. In this study we further investigate changes of the downstream targeting proteins of Nrf2, HO-1 and NQO1, in EMT-induced PF based on our previous study. In Oh’s study, SFN promoting the expressions of Nrf2 and its downstream HO-1 and NQO1 attenuates hepatic fibrosis^30^. Similarly, according to our results, we found expressions of HO-1 and NQO1 were influenced by Nrf2 in EMT-induced PF. When SFN was given on RLE-6TN and A549 cells in vitro, the expressions of HO-1 and NQO1 were augmented with down-regulated α-SMA and up-regulated E-cad. However, in Nrf2 knockout mice or transfecting Nrf2 siRNA in cells, HO-1 and NQO1 were both reduced as well as in BLM-induced model groups and TGF-β1-stimulated groups, which accompanied with the exacerbation of EMT. Experiment results preliminarily suggest that Nrf2, HO-1 and NQO1 are orchestrated to alleviate EMT in PF. But in the present study, we did not knock down HO-1 and NQO1 respectively to block this antioxidant pathway, whether Nrf2 suppressing EMT was completely mediated by HO-1 and NQO1 needs further investigation. At the same time, the effects of other Nrf2 antioxidant target genes, such as superoxide dismutase, glutathione peroxidase still need to be further explored. In the meantime, we discovered the expression of Numb received the regulation of the Nrf2 antioxidant pathway. Nrf2 activator SFN inhibited the expression of Numb under the stimulation of TGF-β1, but in the case of Nrf2 antioxidant pathway being blocked by silenced Nrf2, the protein level of Numb was further augmented compared with TGF-β1 groups, which are consistent with the experiments in vivo. In the fourth part of our experiment, we also briefly detected the inhibitory effect of Nrf2 antioxidant pathway on EMT, which was partly executed by Numb. However, it is still a mystery the role of this regulation being indirect or direct.

**Fig. 8:**
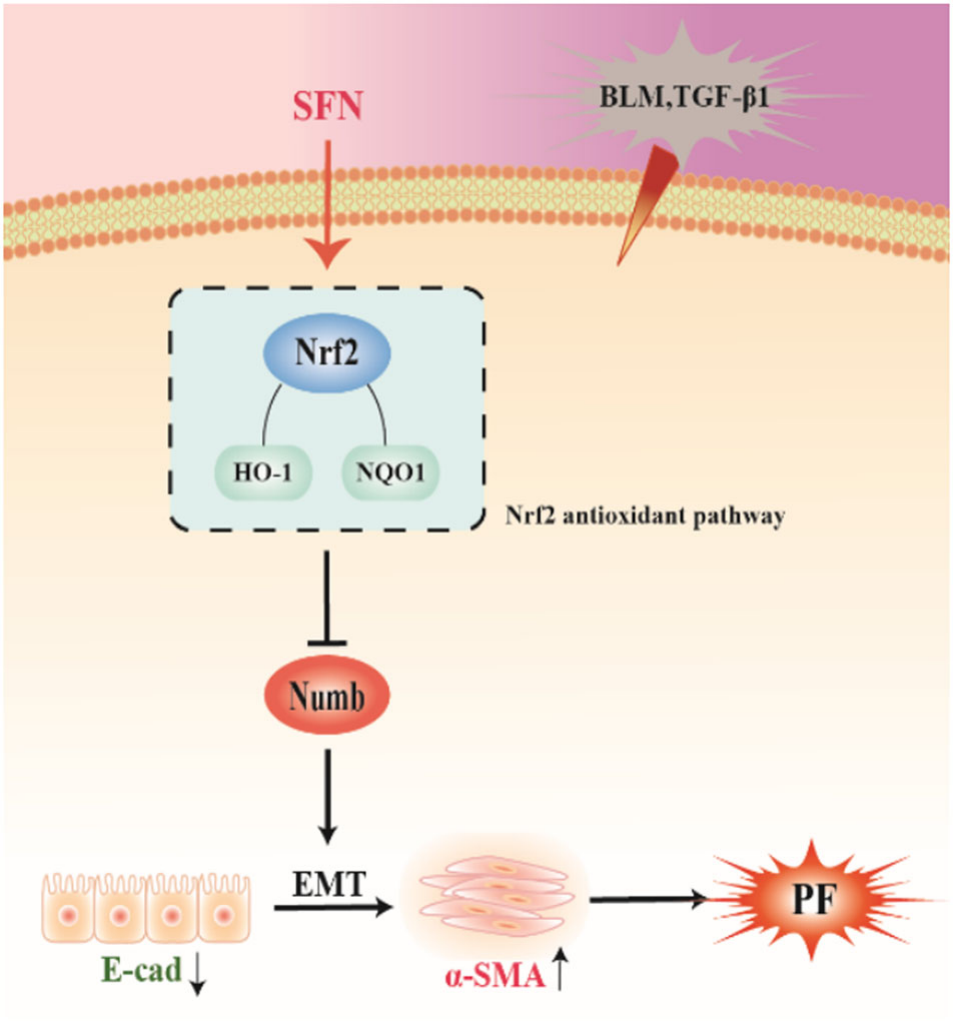
Nrf2 antioxidant pathway protected against EMT-induced PF by suppressing the expression of Numb. Nrf2 antioxidant pathway activated by SFN inhibits Numb-mediated EMT to relieve the progression of PF

In summary, through this research we provide the advanced evidence that Numb involves in EMT-mediated PF, and Nrf2 antioxidant pathway makes an inhibitory role on EMT-mediated PF by suppressing the expression of Numb. Nrf2 pathway and Numb may represent potential therapeutic targets for PF treatment, which need more studies to further investigate (Fig. 8).

## Materials and methods

### Ethics statement

All of the animal procedures involving mice, such as housing and care, and experimental protocols were approved by the Dalian Medical University Animal Care Committee and use committee. All procedures performed on the mice were conducted according to the guidelines from the National Institutes of Health.

### Animal models

Nrf2 knockout mice and their WT littermates were kindly provided by Drs. Peng Cai and teacher Hu (Jiangsu Province Institute of Traditional Chinese Medicine, Nanjing, China), which were originally purchased from the Jackson Laboratory, USA (Order number: 3363093) and fed in the SPF laboratory Experimental Animal Center of Dalian Medical University, Dalian, Liaoning, China. All mice stayed in a specific pathogen-free environment with temperature (23 ± 2 °C),humidity (60 ± 10%) and light cycle (12:12 h light-dark), and were fed a purified diet and water. Then 60 WT mice were randomly assigned into saline group and BLM group (*n* = 30 per group), and sixty Nrf2^−/−^ mice were also randomly divided to two groups (*n* = 30 per group). The PF model was established through intratracheal instillation with 4.5 mg/kg BLM (Laiboten Pharmaceutical CO., LTD, Harbin, China)^14^, while the control group received the same volume of saline instead. On days 7, 14 and 28 after BLM instillation, these mice were anaesthetized with 10% chloral hydrate intraperitoneally, and lung tissues were collected for next experiments.

### Western blot analysis

Mice lung tissue or cells were homogenized in lysis buffer (RIPA; P0013C, Beyotime Institute of Biotechnology, China) mixing with 100 mM proteinase inhibitor phenylmethylsulfonyl-fluoride (PMSF; ST506, Beyotime Institute of Biotechnology, China). Protein was quantified by BCA Protein Assay Kit (Beyotime Institute of Biotechnology, China). After centrifugation (12,000 r/min, 10 min at 4 °C) the supernatant was collected in centrifuged tube, then mixed with loading buffer (Beyotime Institute of Biotechnology, China) according to the ratio of 4:1, heated in dry bath for 10 min, and stored in −20 °C, every sample was tested in 2 weeks. Proteins samples were separated on 12% sodium dodecylsulfate polyacrylaminde gel electrophoresis and transferred onto polyvinyl difluoride membranes (IPVH00010; Millipore, USA). After that the membrane was blocked in 5% non-fat milk for 2 h or over-night, then the membrane was wash by phosphate buffered solution containing Tween20 for three times (each time for 10 min) and incubated with primary antibody at 4 °C overnight, including anti-E-cad (ab76055, abcam), anti-α-SMA (ab5694, abcam), anti-Numb (ab14140, abcam), anti-Nrf2 (ab31163, abcam), anti-HO-1 (ab13243, abcam), anti-NQO-1 (Bioss, bs-2184R) and anti-β-actin (AP0060, bioworld). After 4 °C overnight the membranes were incubated with anti-rabbit IgG (H + L) (Thermo Scientific, Invitrogen, USA) or anti-mouse IgG (H + L) (Thermo Scientific, Invitrogen, USA) for 1 h at room temperature after washing three times (each time for 10 min). Finally, the signals were visualized using the Oddessy Clx (USA), and β-actin was used as an internal reference for relative quantification.

### Histopathologic assessment and immunohistochemical staining

The experiments of Hematoxylin and eosin (H&E), Masson’s trichrome and IHC staining were performed as previously described^24, 31, 32^. Mice lung tissues were fixed by 10% formaldehyde solutions for 24 h, then lung tissues were embedded by paraffin. The paraffin blocks were cut at 5μm using microtome. After that lung tissue slices were stained with Masson’s trichrome staining and H&E staining for histological examination. The expression of Nrf2, Numb, α-SMA and E-cad were investigated by IHC analysis. Biotin-Streptavidin HRP Detection Systems (ZSGB-Bio, China) were used for this experiment and performance steps according to instruction book. Subsequently, lung tissue sections were incubated with primary anti-Nrf2, anti-E-cad, anti-Numb and anti-SMA antibodies at corresponding appropriate ratios of 1:100, 1:250, 1:400 and 1:200 and kept at 4 °C overnight. The next day, the sections were incubated with general secondary antibody working fluid and streptavi din-biotin-peroxidase complex were added to slices for 15 min at 37 °C, and diaminobenzidine was added as a visualizing agent. The nuclear staining with hematoxylin and then using a light microscope (Leica DM2000, Germany) to detect target proteins.

### Cell line and culture

In vitro, rat type II alveolar epithelial cells line (RLE-6TN) was purchased from ATCC (Manassas, USA), and human lung cancer cell (A549) donated by teacher Shao, who works in the Second Affiliated Hospital of Dalian Medical University, all cells grew in DMEM medium (Hyclone, USA) supplemented with 9% fetal bovine serum (FBS; Gibco, USA), the cells were incubated at 37 °C with 5% CO_2_. Cells were incubated with recombinant human TGF-β1 (100–21 C, PeproTech) or SFN (1 μmol/L, Sigma S6317)^14^ in some experiments.

### Transfection

The Nrf2 siRNA, Numb siRNA, and negative control siRNA were designed and synthesized by Gene Pharma (Shanghai, China). The sequences were shown as follows: Nrf2, the forward primer was 5′-GAGGAUGGGAAACCUUACUTT-3′ and the reverse primer was

5′-AUAUUUGCAGUUGAAGGCCTT-3′ used for RLE-6TN. Nrf2, the forward primer was 5′-GCCCAUUGAUGUUUCUGAUTT-3′ and the reverse primer was

5′-AUCAGAAACAUCAAUGGGCTT-3′ used for A549. Numb, the forward primer was 5’-CCAGAAGAUGUCACCCUUUTT-3’ and the reverse primer was 5’-AAAGGGUGACAUCUUCUGGTT-3’used for RLE-6TN. Numb, siRNAsequence was 5’-GGACCTCATAGTTGACCAG-3’used for A549 ^9^.

### Immunofluorescence

The cells were fixed with 4% paraformaldehyde for 20 min, and then washed with PBS and permeabilization with 0.1% Triton-X 100. After blocking with 5% BSA for 30 min at room temperature, the cells were incubated with the anti-Numb antibody (1:150) at 4 °C overnight. Cells were rinsed with PBS for three times, and added with FITC-conjugated goat anti-mouse IgG (ZF-0311; Beijing, China, 1:200) for 1 h. 6-diamidino-2-phenylindole (DAPI) (C1005; Beyotime Institute of Biotechnology, Shanghai, China) was used to identify the nucleus. The images were captured by an Olympus UTBI90 Fluorescence microscope with the appropriate filters and identical acquisition parameters at ×100 magnification.

### Statistical analysis

All data are expressed by mean ± standard deviation (SD), using social science statistical software package for data analysis. The two groups of data were compared with the independent sample *t* test and comparison of multiple sets of data using a one-way analysis of variance analysis. The column graphs were drawn using graphpad prism6 software. *P* < 0.01 or *P* < 0.05 had statistical significance.
